# Mouse Cytomegalovirus *M34* Encodes a Non-essential, Nuclear, *Early*-*Late* Expressed Protein Required for Efficient Viral Replication

**DOI:** 10.3389/fcimb.2020.00171

**Published:** 2020-05-05

**Authors:** Mareike Eilbrecht, Vu Thuy Khanh Le-Trilling, Mirko Trilling

**Affiliations:** Institute for Virology, University Hospital Essen, University of Duisburg-Essen, Essen, Germany

**Keywords:** cytomegalovirus, M34, UL34, non-essential, BAC mutagenesis

## Abstract

Human cytomegalovirus (HCMV) is a prototypic betaherpesvirus which causes severe manifestations in individuals with impaired or immature immunity. To investigate cytomegalovirus-induced pathogenesis and virus-specific immune responses, mouse cytomegalovirus (MCMV) infections in mice are employed as accepted small animal model. MCMV and HCMV share co-linear genomes and encode several homologous proteins. Due to the size and complexity of CMV genomes, the molecular functions of numerous cytomegaloviral gene products remain to be elucidated. While the essential nature of viral genes highlights their biological relevance, it renders functional studies particularly cumbersome by precluding experiments in the infection context. The HCMV-encoded protein pUL34 binds the HCMV genome and regulates viral gene expression (e.g., of *US3*). Several groups provided compelling evidence that *UL34* is essential for HCMV replication. MCMV encodes the homologous protein pM34 (34% identical and 55% similar). Based on unsuccessful attempts to reconstitute *M34*-deficient virus from a bacterial artificial chromosome (BAC), *M34* was previously classified as essential for MCMV replication. To characterize pM34 during viral infection, we engineered and analyzed an MCMV mutant expressing an HA-epitope-tagged pM34 which was expressed with *early-late* kinetics and localized in the nucleus. Additionally, we generated an *M34*-deficient (“ΔM34”) MCMV-BAC by replacing the entire *M34* coding sequence by a kanamycin resistance cassette. The deletion of *M34* was confirmed by Southern blot and PCR. Unexpectedly, we could reconstitute replicating ΔM34-MCMV upon transfection of the BAC DNA into mouse embryonic fibroblasts. The absence of *M34* from the genome of the replicating ΔM34-MCMV was also confirmed. Accordingly, a ΔM34-MCMV, in which the kanamycin cassette was excised by *frt*/Flp-mediated recombination, was also replication competent. In order to corroborate the absence of pM34 protein, the *M34* deletion was recapitulated on the background of M34HA, which yielded replicating virus devoid of detectable pM34HA protein. The replication of MCMVs lacking *M34* was found to be 10- to 100-fold reduced as compared to wt-MCMV which might explain previous unsuccessful reconstitution attempts conducted by others. Taken together, our findings reveal that MCMV remains replication competent despite the absence of *M34*, enabling functional studies in the infection context.

## Introduction

More than half of the global human population is infected with the *Human cytomegalovirus* (HCMV; also called *Human herpesvirus 5* [HHV5]; Taxonomy ID [TaxID]: 10359). HCMV infections are usually subclinical in healthy adults, and fatal infection in apparently immunocompetent individuals are rare (Rafailidis et al., 2008). Nevertheless, even the uncompromised adult immune system is incapable to completely eliminate HCMV. Although HCMV infections are usually confined and controlled by a concerted action of all branches of the immune system, thereby alleviating or ideally preventing HCMV-induced diseases, residual replication-competent virus inevitably remains life-long in a dormant state called latency. Once the host experiences stress or immune-compromising conditions, HCMV can reactivate from latency leading to recurrent diseases. Individuals with an immature, compromised, or senescent immune system often fail to control HCMV replication. Depending on the degree and duration of impaired immunity, HCMV causes mortality and morbidity under such conditions. Accordingly, congenitally infected infants, transplant recipients, and HIV-infected AIDS patients are prone to life-threatening HCMV infections. Consistent with the fact that HCMV can replicate in a variety of different cell types and tissues, it can elicit a broad spectrum of clinical syndromes such as sensorineural hearing loss and mental retardation in congenitally infected infants, retinitis in HIV/AIDS patients, and pneumonia in transplant patients.

HCMV is the prototypical member of the *Betaherpesvirinae* subfamily of herpesviruses. Resulting from millions of years of co-evolution, cytomegaloviruses (CMVs) specifically adapted to their corresponding host species. Although cross-species infections may occur and contribute to the evolution of CMVs (Murthy et al., 2019), CMV species are usually restricted to one or few closely related host species *in natura*. In agreement with the species specificity, HCMV is incapable to productively replicate in small animals such as mice or rats. A notable exception are humanized mice, in which HCMV replicates in engrafted human tissues (Smith et al., 2010). The inability to perform defined HCMV infection experiments in small animal models constitutes a relevant hurdle for research. Therefore, mouse cytomegalovirus (MCMV, *Murid herpesvirus 1* [MuHV1], TaxID: 10366) has been established and is commonly used as a small animal model for studying general principles of CMV infection and pathogenesis *in vivo* (Brizić et al., 2018). Additionally, MCMV is one of the few viruses infecting *Mus musculus* as genuine host species, enabling research on a natural virus-host interaction. Thus, MCMV has become a standard model for immunology which helped to uncover fundamental principles of immunity such as cytotoxic CD4+ lymphocytes (Jonjic et al., 1990), NK cell memory (Sun et al., 2009), or T cell memory inflation (Holtappels et al., 2000; Karrer et al., 2003). HCMV and MCMV have very large co-linear double-stranded DNA genomes of more than 230 kb. CMV genomes are very complex, comprising genes located on both DNA strands, the use of alternate start codons, and alternative splicing events, resulting in the case of HCMV in more than 750 translation products (Stern-Ginossar et al., 2012; Erhard et al., 2018). Certain protein families are either conserved between all *Herpesvirales, Betaherpesvirinae*, or CMVs (see e.g., Rawlinson et al., 1996). Despite their importance indicated by conservation, numerous of these proteins are far from being fully understood. A fraction of gene products (e.g., the viral DNA polymerase or glycoprotein B) fulfill indispensable functions during the viral replication cycle. The corresponding genes are referred to as essential genes. Mutant genomes lacking an essential gene cannot be reconstituted on non-complementing cells. Additionally, there is a class of semi-essential genes such as *IE1*, which are essential under low MOI infection conditions, but can be partially compensated by other gene products under higher MOI conditions (Mocarski et al., 1996). While the essential nature highlights their relevance, it also renders the investigation of such genes particularly difficult due to the impossibility to conduct infection experiments. One option to circumvent this issue are conditional knock-out systems, which either induce or ablate the protein of interest under defined conditions such as addition of a (de-) stabilizing drug (see e.g., Glass et al., 2009). Another approach is to study homologous proteins expressed by related viruses.

Several groups have shown that the gene *UL34* is essential for HCMV replication (Dunn et al., 2003; Yu et al., 2003; Rana and Biegalke, 2014). Biegalke et al. described two predominant and a less-abundant gene product derived from *UL34* during the course of infection (Biegalke et al., 2004; Rana and Biegalke, 2014). The pUL34 proteins are enriched in the nucleus where they bind DNA and regulate the expression of viral genes (e.g., *US3* and *US9*) through transcriptional repressor functions (LaPierre and Biegalke, 2001; Liu and Biegalke, 2013). The HCMV genome of strain AD169 contains 14 pUL34 consensus binding sites (consensus motif AAACACCGT[G/T]), three of which reside in the region of the *origin of lytic replication* (*oriLyt*) (Liu and Biegalke, 2013). Chromatin immunoprecipitation (ChIP) experiments confirmed the binding of pUL34 to the predicted sites in the *oriLyt* and identified another binding site in the *oriLyt* harboring the similar but non-identical sequence motif AAACgCCGTc (Slayton et al., 2018). Site-directed mutagenesis of individual pUL34 binding sites within the *oriLyt* significantly attenuated HCMV replication (Slayton et al., 2018), suggesting a relevant role of pUL34-*oriLyt* associations. However, a mass spectrometry (MS)-based assessment of proteins associated with cell-free HCMV virions did not identify pUL34 as constituent of the HCMV particle (Varnum et al., 2004), implying that pUL34 may be stripped of the HCMV genome before or during packaging. Additionally, pUL34 physically interacts with pIE2, pUL44, and pUL84 (Slayton et al., 2018).

MCMV encodes the homologous protein pM34 (34% identity and 55% similarity). *M34* mRNA expression has been documented in infected NIH/3T3 cells by an MCMV microarray (Tang et al., 2006). To our knowledge, the function of pM34 has not been addressed so far. Similar to pUL34, pM34 was not detected in the MCMV virion (Kattenhorn et al., 2004). Comprehensive analyses failed to identify pM34-specific CD8+ and CD4+ lymphocytes (Munks et al., 2006; Walton et al., 2008). Our MS-based analysis showed that pM34 is expressed and that its abundance is significantly reduced upon treatment with MLN4924 (Le-Trilling et al., 2016). Baluchova et al. reported that an *M34*-deficient MCMV could not be reconstituted from a bacterial artificial chromosome (BAC) (Baluchova et al., 2008). This was interpreted as an indication that *M34*, similar to *UL34*, is essential for viral replication. In contrast, an *M34* transposon insertion mutant as well as a truncation mutant, both expressing a C-terminally truncated pM34 form containing the most conserved region, were reported to be in principle replication competent *in vitro*, albeit with reduced replication efficiency (Baluchova et al., 2008).

Here, we constructed an MCMV mutant expressing an HA-epitope-tagged pM34 to characterize pM34-HA during viral infection. Additionally, we generated *M34*-deficient (“ΔM34”) MCMV-BACs. Contradicting previous reports, we successfully reconstituted replicating ΔM34-MCMV in non-complementing cells, documenting that MCMV is attenuated, yet replication competent *in vitro* despite the absence of *M34*.

## Materials and Methods

### Cells, Culture Media, and Inhibitors

Primary mouse embryonic fibroblasts (MEF) from C57BL/6 mice and mouse newborn cells (MNCs) from BALB/c mice were isolated as described before (Le-Trilling and Trilling, 2017). For all experiments, MEF and MNC were used in passage 3. A stable MEF cell line derived from C57BL/6 embryos (CIM [“*C*57BL/6 *i*mmortalized *M*EF”]) had been generated by crisis immortalization (Rattay et al., 2015). All cells were cultured in Dulbecco's minimal essential medium (DMEM) supplemented with 10% (v/v) FCS, 100 μg/ml streptomycin, 100 U/ml penicillin, and 2 mM glutamine (Gibco/Life technologies). Cycloheximide, Actinomycin D, and phosphonoacetic acid were purchased from Roth, AppliChem, and Sigma-Aldrich, respectively.

### Viruses and Infections

#### Generation of Virus Mutants by BAC Mutagenesis

For the generation of recombinant MCMV mutants, the *mck2*-repaired MCMV-BAC (Jordan et al., 2011) was used. For the addition of a C-terminal HA-tag to the *M34* ORF by *en passant* mutagenesis (Tischer et al., 2006), a PCR fragment was generated using the primer C3X-M34HA-1 (CCGCCCAGAAACATTCTGAGTGCATCAACATCCTGCTCTACCCATACGATGTTCCAGATTACGCTTAAGGGGGGCGCGGGACGaggatgacgacgataagtaggg), the universal Kana S primer (CAACCAATTAACCAATTCTGA), and the Kana S plasmid as the template DNA. The amplified fragment served as template for a second PCR, applying the primers C3X-M34HA-2 (CGTGTCTCGACCGTTCCCCTCGTCCCGCGCCCCCCTTAAGCGTAATCTGGAACATCGTATGGGTAGAGCAGGATGTTGATGCAcaaccaattaaccaattctgattag) and C3X-M34HA-3 (CCGCCCAGAAACATTCTGAG). The resulting amplificate containing the kanamycin resistance gene was inserted into the MCMV-BAC by Redαβ-mediated homologous recombination in GS1783 *E. coli* cells (Tischer et al., 2010), followed by the deletion of the resistance marker by a I-S*ce*I-mediated cleavage and a homologous recombination event. ΔM34-MCMV harboring a deletion of the entire *M34* ORF was generated by amplifying an *frt*-site-flanked kanamycin cassette using the plasmid pFRT1 as the template DNA and the primers ΔM34-Kana1 (CAGCGGTGCTACGCATCACCTCAGACGCCGCGCCGCCGCCACTAACAGTTTGCTCGCTCGCCAGTGAATTCGAGCTCGGTAC) and ΔM34-Kana2 (GTGAGGGAGACGGTGTCGCGGACGCCGTGTCTCGACCGTTCCCCTCGTCCCGCGCCCCCCGACCATGATTACGCCAAGCTCC). The PCR fragment was introduced into the MCMV-BAC by Redαβ-mediated homologous recombination in GS1783 *E. coli* cells, replacing the *M34* ORF by the kanamycin resistance gene. Afterwards, the *frt*-site-flanked kanamycin cassette was deleted from the BAC by Flp-mediated recombination in DH10B *E. coli* cells. To verify the mutations, the altered sequences were amplified by PCR using the ΔM34^KanaR−^-MCMV-BAC and the M34HA MCMV-BAC as template (see **Figure 2**). The PCR products were send for sequencing by LGC Genomics (Berlin, Germany). The sequencing results confirmed that the deletion/insertion occurred as intended (data not shown).

M34HA-MCMV and ΔM34-MCMV were reconstituted by transfection of BAC DNA into CIM using Superfect (Qiagen). Transfected cells were cultured until plaque formation was observed and passaged until sufficient viral yields were reached for preparation of seeding stocks. MCMV stocks were prepared on CIM. Cells were infected with centrifugal enhancement (900 g, two times for 15 min), and passaged until all cells showed cytopathic effects (CPE). Infected cells and supernatants were harvested, and crude stocks or purified stocks were prepared as described elsewhere (Brune et al., 2001). Virus titers were determined by standard plaque titration assay as described previously (Brune et al., 2001).

#### Infections and Plaque Titration Assay

All infections were performed with centrifugal enhancement (900 g, two times for 15 min). Viral DNA replication was inhibited with 250 μg/ml phosphonoacetic acid (PAA; Sigma-Aldrich). PAA was administered 25 min prior to infection and retained until the end of the experiment (48 h p. i.). For replication analysis, infected cells were frozen at 1, 3, and 4 or 5 days post infection. Viral titers were determined by standard plaque titration (Brune et al., 2001) on MEF or MNC. MCMV replication experiments were independently conducted at least three times. All titrations were performed in triplicate.

### Southern Blotting and PCR

BAC DNA was cleaved using *Eco*RI (New England Biolabs). DNA fragments were separated by gel electrophoresis and transferred to nylon membranes by capillary blotting. Probes were generated by PCR using the following primer sets: (I) ΔM34-ctrl1 (CAGAGACGCTACTCTGATCGC) and ΔM34-ctrl2 (GAGGTCTCGGTTCTTCTCCAC), (II) M33-1 (GACGGATCCATGGACGTCCTTTTGGGCCGG) and M33-2 (GCATCTCGAGTCAAGCGTAATCTGGAACATCGTATGGGTACTGGGGCGGAGGAGCGC), (III) m157-1 (GTCATCGTCCCCCTAGTAAAATTAC) and m157-2 (GTCGAACTGACATCCGGACAG), and (IV) M27-forw (AGCCCTTTAATCACATCGAA) and M27-rev (TGAAGTAGACGTTGTTGGCC). The hybridization and detection were performed following manufacturer's (Roche) instructions.

For PCR controls, DNA of infected CIM served as template for PCRs using the following primers: ΔM34-ctrl1 (CAGAGACGCTACTCTGATCGC), ΔM34-ctrl2 (GAGGTCTCGGTTCTTCTCCAC), MCMV IE1-1a (GAGCCCGCCGCACCCAGGG), MCMV IE1HA-2 (CG*GAATTC*TCAAGCGTAATCTGGAACATCGTATGGGTACTTCTTGCTCTTCTTCTTGGGC), Kana-ctrl1 (CAACAAAGCCACGTTGTGTCTC), and Kana-ctrl2 (CCATAGGATGGCAAGATCCTGG).

### Western Blotting

For immunoblotting, cells were lysed as described before (Trilling et al., 2009) and protein concentrations were adjusted according to Bradford protein assays. Whole cell lysates were subjected to SDS polyacrylamid gel electrophoresis (SDS-PAGE) and subsequently transferred onto nitrocellulose membranes. Immunoblot analysis was performed using mouse antibodies anti-Actin (A2228, Sigma-Aldrich), anti-pp89 (CROMA101, Center for Proteomics, Rijeka, Croatia), anti-M55/gB MCMV (15A12-H9, Center for Proteomics, Rijeka, Croatia), and rabbit antibodies anti-HA (H6908, Sigma-Aldrich) and anti-GAPDH (sc-24778, Santa Cruz). Proteins were visualized using peroxidase-coupled secondary antibodies (115-035-062, ImmunoResearch, and A6154, Sigma-Aldrich) and the substrate SignalFire ECL reagent (Cell Signaling Technology).

### Cloning of Expression Vectors

For cloning of pcDNA3.1-UL34HA, UL34HA was amplified by use of the primers UL34-1 (CCGCTCGAGATGAACTTCATCATCACCACCC) and UL34HA-2 (CGCGGATCCTTAAGCGTAATCTGGAACATCGTATGGGTAAATACACAACGGGGTTATG). The product was introduced into pMiniT and subcloned into pcDNA3.1. The CDS of *M34HA* was cloned into the pcDNA3.1 vector using the In Fusion HD Cloning Kit (Takara) according to manufacturer's instructions. *Bam*HI and *EcoR*I restriction sites and a C-terminal HA epitope tag were introduced by specific primers M34-For (TACCGAGCTCGGATCCATGGAAACCGCGTCGGCTTCT) and M34HA-Rev (GATATCTGCAGAATTCTTAAGCGTAATCTGGAACATCGTATGGGTAGAGCAGGATGTTGATGCACTCAG). The inserts were sequenced at LGC Genomics (Berlin, Germany).

### Immunofluorescence Microscopy

HeLa cells were transfected with pcDNA3.1-UL34HA or pcDNA3.1-M34HA using Superfect (Qiagen) according to manufacturer's instructions. CIM cells were infected with wt-MCMV or M34HA-MCMV (MOI 0.7) or left uninfected. Twenty-four h post transfection or infection, cells were fixed with 4% paraformaldehyde and permeabilized with 0.02 % Triton X-100. Blocking was conducted with 2% FCS for transfected cells and 2% mouse serum for infected cells. Subcellular localization of M34HA and UL34HA was visualized by immunostaining using rabbit primary antibody anti-HA (H6908, Sigma-Aldrich) and secondary antibody anti-rabbit-Cy2 (115-225-146, Dianova). DNA was stained with DAPI (Sigma-Aldrich). Microscopy was conducted with a Leica DM IL LED Microscope and the LAS V4.0 software (Leica).

### BLAST

The pM34 amino acid sequence of the MCMV reference strain (MuHV1 YP_214047.1) was blasted against Herpesviridae (Herpesviridae, TaxID:10292, reference proteins) using NCBI Blastp. Clustal Omega (https://www.ebi.ac.uk/Tools/msa/clustalo/) was used for pairwise and multiple alignments.

### Statistical Analysis

The significance of differences among the replication of two virus mutants was analyzed by two-tailed heteroscedastic *t*-test. For replication analysis of three virus mutants, two-way ANOVA with multiple comparisons was applied.

## Results

### pM34, the MCMV-Encoded Homolog of pUL34, Is Expressed With *Early/Late* Kinetics and Is Localized in the Nucleus

**Figure 1:**
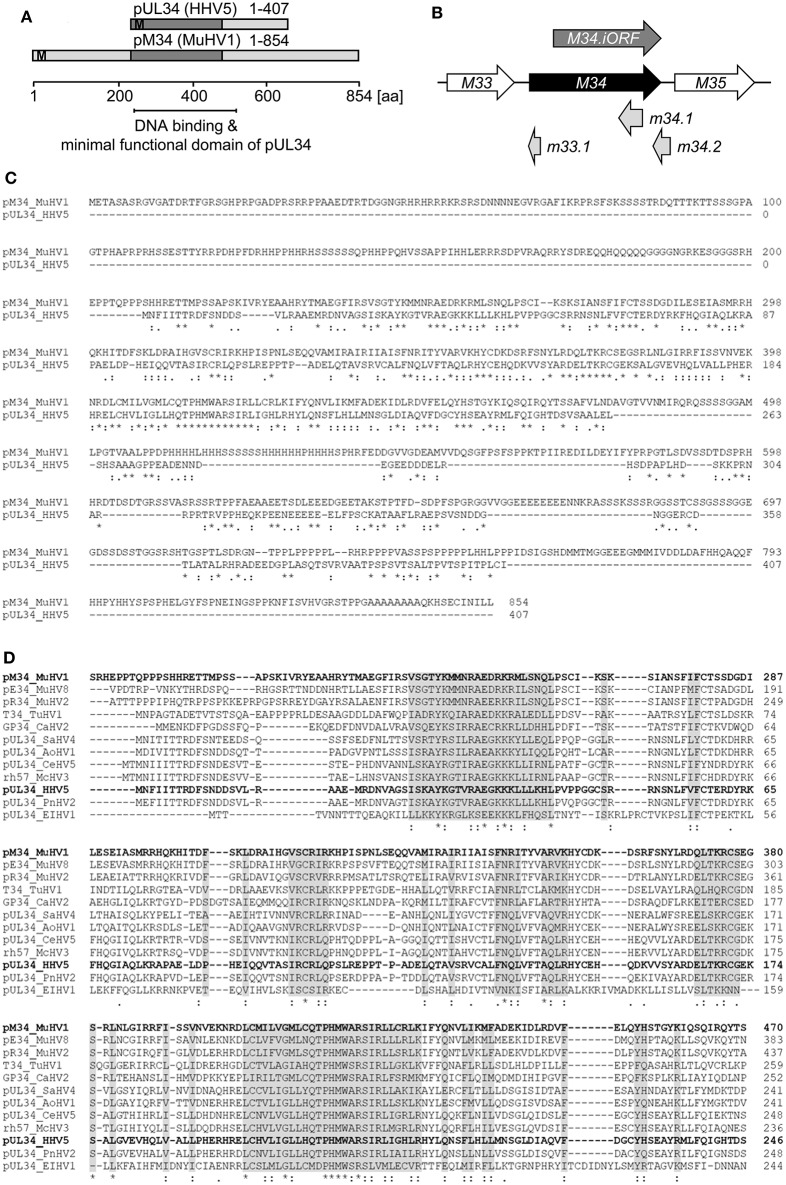
Alignment of HCMV- and MCMV-encoded homologous proteins pUL34 and pM34. **(A)** Schematic overview of the conserved regions (dark gray) of pUL34 (HHV5/HCMV) and pM34 (*Murid betaherpesvirus* [MuHV] 1/MCMV). M: methionine. **(B)** Schema of the MCMV ORF *M34* (black) and the neighboring ORFs *M33* and *M35* (white) according to the annotation of Rawlinson et al. (1996). The more recently predicted ORFs m33.1, m34.1 (Brocchieri et al., 2005) and m34.2 (Tang et al., 2006) are depicted in light gray. A truncated internal in-frame ORF, M34.iORF, predicted by Brocchieri et al. is depicted in dark gray (Brocchieri et al., 2005). **(C)** Alignment of pM34 (MuHV1/MCMV) and pUL34 (HHV5/HCMV) using Clustal Omega (https://www.ebi.ac.uk/Tools/msa/clustalo/). **(D)** The pM34 protein sequence of the MCMV reference strain (pM34 MuHV1 YP214047.1) was blasted against Herpesviridae (Herpesviridae [TaxID: 10292] reference proteins) using NCBI Blastp. A multiple alignment of the most conserved proteins in cytomegaloviruses is shown (Clustal Omega). Sequences are ordered in decreasing identity (%) as obtained by Blastp. pM34 and pUL34 are marked in bold. pM34_MuHV1, pE34_MuHV8: *Murid betaherpesvirus 8* (TaxID 1261657), pR34_MuHV2: *Murid betaherpesvirus 2* (TaxID 28304), T34_TuHV1: *Tupaiid betaherpesvirus 1* (TaxID 10397), GP34_CaHV2: *Caviid betaherpesvirus 2* (TaxID 33706), pUL34_SaHV4: *Saimiriine betaherpesvirus 4* (TaxID 1535247), pUL34_AoHV1: *Aotine betaherpesvirus 1* (TaxID 50290), pUL34_CeHV5: *Cercopithecine betaherpesvirus 5* (TaxID 50292), rh57_McHV3: *Macacine betaherpesvirus 3* (TaxID 47929), pUL34_HHV5, pUL34_PnHV2: *Panine betaherpesvirus 2* (TaxID 188763), pUL34_EIHV1: *Elephantid betaherpesvirus 1* (TaxID 146015).

Proteins exhibiting significant homology to the HCMV-encoded pUL34 can be identified in several cytomegaloviruses infecting primates, rodents, and other genera. We failed to identify proteins with significant homology to pUL34 beyond the clades of Cytomegaloviruses and Muromegaloviruses, e.g., in *Alpha*- or *Gammaherpesvirinae* (data not shown). An amino acid sequence alignment revealed that pUL34 and its homolog pM34 share a conserved central domain (see schematic overview in Figure 1A). In addition to the canonical *M34* predicted by Rawlinson et al. (1996), a truncated internal in-frame ORF (iORF) was suggested by Brocchieri et al. (2005) (Figure 1B). The highly conserved region present in pM34 and pM34.iORF overlaps with the domains described to confer DNA binding, transcriptional regulatory capacity, nuclear localization, and nuclear retention of pUL34 (Biegalke et al., 2004; Biegalke, 2013), suggesting functional homologies of this protein family. The MCMV-encoded protein pM34 is 34% identical and 55% similar to pUL34 (Figure 1C). In contrast to primate CMVs, Muromegaloviruses such as MCMV, *Murid herpesvirus 2* (Rat cytomegalovirus “*Maastricht*”), and Murid herpesvirus 8 (Rat cytomegalovirus “*England*”) possess N-terminally extended sequences, suggesting additional lineage-specific adaptations (Figure 1D).

**Figure 2:**
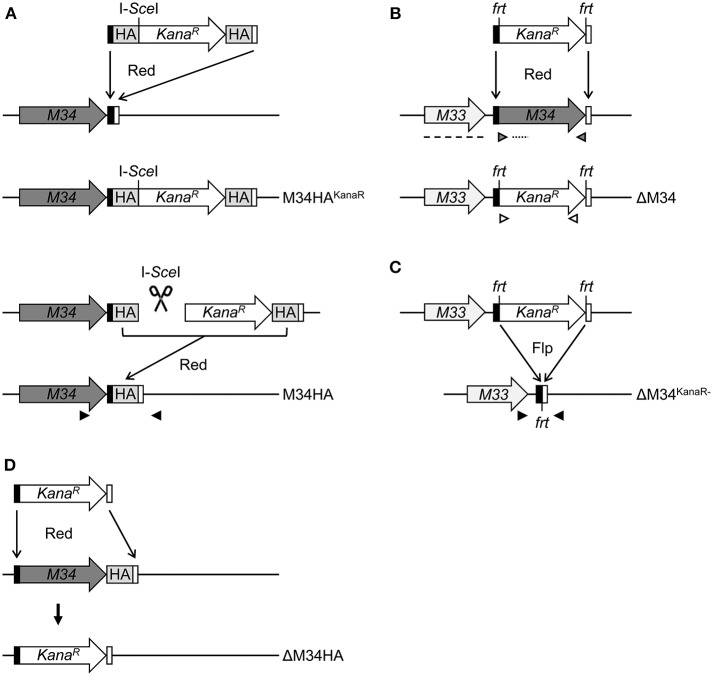
Schematic overview of MCMV-BAC mutagenesis. **(A)** Generation of M34HA-MCMV by insertion of an HA-epitope tag by *en passant* mutagenesis (see methods section for details). **(B)** Generation of a virus mutant harboring a deletion of the *M34* ORF (ΔM34-MCMV) by replacement with a kanamycin cassette. **(C)** Excision of the kanamycin cassette from the ΔM34-MCMV BAC (ΔM34^KanaR−^) by *frt*/Flp-mediated recombination. **(D)** Generation of a virus mutant harboring a deletion of the *M34HA* ORF (ΔM34HA) by replacement of M34HA with a kanamycin cassette. M34, *M34* ORF (gray); KanaR, kanamycin resistance gene (white); black boxes, 5′ homologous region; white boxes, 3′ homologous region; HA, HA-epitope tag (light gray); I-S*ce*I, I-S*ce*I restriction site; *frt*, flippase recognition target; Flp, flippase; Red, Red recombinase derived from the λ bacteriophage. Black arrow heads: sequencing primers, gray arrow heads: M34 PCR control primers, white arrow heads: KanaR PCR control primers, dotted line: M34 southern blot control probe, dashed line: M33 southern blot control probe.

**Figure 3:**
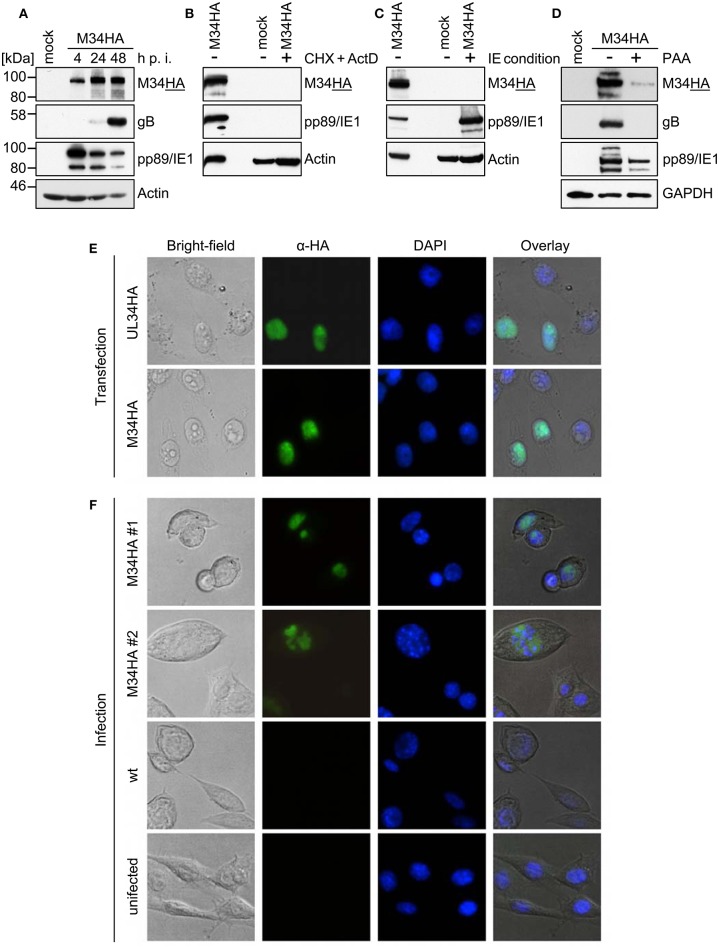
pM34HA is expressed with *early/late* kinetics and is localized in the nucleus. **(A)** CIM cells were infected with M34HA-MCMV (MOI 10) or left uninfected (mock). At indicated time points post infection, cells were lysed and immunoblot analysis of indicated proteins was performed. **(B)** CIM were briefly pre-incubated and then infected with M34HA-MCMV (MOI 10) in the presence and absence of 50 μg/ml Cycloheximid (CHX) and 5 μg/ml Actinomycin D (Act D) or left untreated and uninfected. Lysates were prepared after 4 h and analyzed by immunoblot. **(C)** CIM were infected with M34HA-MCMV (MOI 10) in the presence and absence of CHX or left untreated and uninfected. After 4 h, CHX was replaced by Act D and incubated for 4 h. Lysates were analyzed by immunoblot using antibodies specific for indicated proteins. **(D)** CIM cells were treated with 250 μg/ml phosphonoacetic acid (PAA) for 25 min prior to infection or left untreated. Cells were infected with M34HA-MCMV (MOI 10) for 48 h in the absence or presence of PAA or left uninfected (mock) and analyzed by immunoblot using antibodies specific for indicated proteins. **(E)** HeLa cells were transfected with pcDNA3.1-UL34HA or pcDNA3.1-M34HA. Twenty-four hour post transfection, cells were fixed and immunofluorescence staining was conducted using rabbit anti-HA and anti-rabbit-Cy2. DAPI staining was included for visualization of nuclei. **(F)** CIM cells were infected with wt-MCMV, M34HA-MCMV (MOI 0.7), or left uninfected. Cells were fixed at 24 h post infection. Immunofluorescence staining was performed as described above.

To study the protein expression of pM34 in the viral context, we inserted a sequence coding for an HA-epitope tag directly between the end of the coding sequence (CDS) and the Stop codon of *M34* into the MCMV genome by *en passant* BAC mutagenesis (Figure 2). The correct insertion of the HA-epitope sequence was confirmed by sequencing of the PCR-amplified *M34* ORF (data not shown) and by immunoblotting of whole cell lysates of infected cells using HA-specific antibodies (Figure 3A). One prominent pM34HA protein of approximately 90-100 kDa was detectable at 4 h post infection and the abundance further increased until 24 h post infection (Figure 3A). The molecular weight of the detected pM34HA protein was consistent with the prediction of 94.5 kDa by Rawlinson et al. (1996). When the infection was conducted in the presence of Cycloheximide (CHX) and Actinomycin D (ActD) to prevent viral gene expression, pM34HA was undetectable (Figure 3B), consistent with the notion that it is not part of the incoming virion. This finding is consistent with previous data (Kattenhorn et al., 2004). Under selective IE expression conditions [by infection in the presence of CHX which was washed out at 4 h p. i. in the presence of ActD followed by 4 h in presence of ActD; see (Rattay et al., 2015)], pp89/IE1 was detectable, while pM34HA was not (Figure 3C). Upon addition of the antiviral drug phosphonoacetic acid (PAA), which prevents viral genome replication and impairs viral *late* gene expression, pM34HA expression was diminished but not abrogated (Figure 3D). The latter result is in agreement with previously published mRNA data (Chapa et al., 2014). Together these findings indicate that pM34 is expressed in MCMV-infected cells with *early/late* kinetics.

In order to compare the subcellular localization of pM34 with pUL34, cells were transfected with expression plasmids encoding HA-epitope tagged versions of these proteins and analyzed by immunofluorescence staining. In agreement with previous publications (Rana and Biegalke, 2014), pUL34 was detected in the nucleus (Figure 3E). A similar localization was observed for pM34HA upon transfection (Figure 3E) as well as upon MCMV infection (Figure 3F).

### Generation of ΔM34-MCMV by BAC Mutagenesis

**Figure 4:**
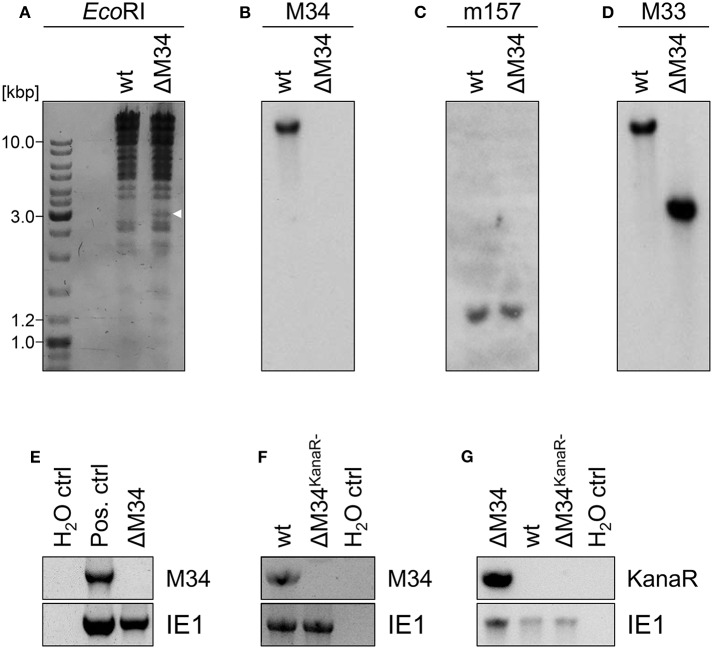
Validation of ΔM34-MCMV. **(A–D)** BAC DNA of wt and ΔM34 was used for validation of the *M34* deletion. **(A)** BAC DNA was digested with E*coR*I. The restriction fragments were separated by gel electrophoresis. The white arrow marks the additional fragment at 3 kbp of ΔM34-MCMV BAC. The specific deletion of the *M34* ORF from the BAC was confirmed by Southern blot using probes specific for **(B)** M34, **(C)** m157, and **(D)** M33. **(E)** The absence of *M34* from the genome of ΔM34-MCMV was confirmed by PCR using DNA extracted from infected cells used for virus stock preparation as template. **(F,G)** The deletion of *M34* and the kanamycin cassette from the genome of ΔM34^KanaR−^-MCMV was confirmed by PCR of DNA extracted from infected cells used for virus stock preparation.

To generate an MCMV-BAC harboring a deletion of *M34*, a PCR fragment containing a kanamycin resistance gene was introduced into an *mck2*-repaired MCMV-BAC replacing the entire *M34* ORF (Figure 2B). Consistent with an *in silico* prediction (2797 bp), a restriction fragment analysis of the mutated BAC using *Eco*RI revealed an additional fragment of approximately 3 kbp compared to a parental BAC control (Figure 4A; highlighted by white arrowhead). The correct replacement of the *M34* ORF by the kanamycin resistance gene was further confirmed by Southern blotting. The specific signal recognized by an *M34*-specific probe was lost in ΔM34-MCMV BAC DNA (Figure 4B), while *m157* remained detectable (Figure 4C). Furthermore, an *M33*-specific probe confirmed that the insertion of the resistance cassette did not inadvertently delete the neighboring *M33* ORF (Figure 4D), but changed the size of the corresponding *Eco*RI restriction fragment (Figures 4A,D).

Contrary to our expectations based upon previous reports (Baluchova et al., 2008), plaque formation was observed after transfection of the ΔM34-MCMV BAC into CIM cells and a couple of ‘blind passages’, in which no CPE or plaque formation was apparent. First plaques were observed after 17 days. A crude seeding stock of ΔM34-MCMV was harvested at day 24 after reconstitution. To exclude contaminations, the deletion of *M34* from the genome of replicating ΔM34-MCMV was controlled by PCR using DNA extracted from infected cells as template (Figure 4E). To formally rule out that the insertion of the kanamycin resistance gene affects for example neighboring genes, the cassette was deleted by Flp-mediated recombination (Figure 2C). Correct deletion of the entire *M34* coding sequence and excision of the kanamycin cassette were confirmed by sequencing of the specific region of the ΔM34^KanaR−^-MCMV-BAC (data not shown). Reconstitution of ΔM34^KanaR−^-MCMV in CIM cells was successful (data not shown). The absence of *M34* and the deletion of the kanamycin cassette were confirmed by PCR using DNA extracted from infected cells used for stock preparation as template (Figures 4F,G). The successful reconstitution of replicating virus from BAC DNA harboring a complete deletion of the *M34* coding sequence, irrespective of the presence or absence of the kanamycin cassette, demonstrates that *M34* as well the overlapping genes *m33.1* and *m34.1* (see Figure 1B) are not essential for MCMV replication.

### ΔM34-MCMV Is Replication Competent but Attenuated *in vitro*

**Figure 5:**
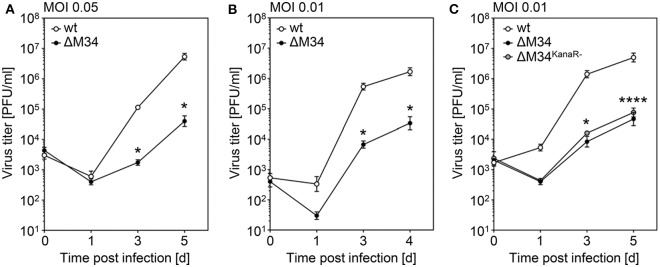
ΔM34-MCMV is replication competent but highly attenuated *in vitro*. **(A)** CIM cells were infected with wt-MCMV or ΔM34-MCMV (MOI 0.05). At indicated time points post infection, virus titers were quantified by standard plaque assay. ^*^p < 0.05 (two-tailed *t*-test, unpaired). **(B)** Primary MNC were infected with wt-MCMV or ΔM34-MCMV (MOI 0.01). Virus titers were quantified by standard plaque assay. ^*^p < 0.05 (two-tailed *t*-test, unpaired). **(C)** CIM were infected with wt-MCMV, ΔM34-MCMV, or ΔM34^KanaR−^-MCMV (MOI 0.01). Virus titers were quantified after indicated time points post infection. ^*^p < 0.05. ^****^p < 0.0001. Significance was calculated by two-way ANOVA with multiple comparisons. Each titration was performed in triplicate and depicted as mean values ± standard deviation (SD).

To determine the role of the *M34* ORF for MCMV replication, CIM cells were infected with wt-MCMV or ΔM34-MCMV at low MOI (0.05 PFU/cell). At 1, 3, and 5 days post infection, aliquots were collected and cryo-conserved. MCMV titers were quantified by standard plaque titration on MEFs. In several independent experiments, the replication of ΔM34-MCMV was 10- to 100-fold reduced as compared to wt-MCMV (see Figure 5A and data not shown). The replication of ΔM34-MCMV was also impaired in primary mouse newborn cells (MNC; MOI 0.01) isolated from BALB/c mice, indicating a cell type and mouse strain independent replication phenotype *in vitro* (Figure 5B). The attenuation was evident irrespective of the presence or absence of the kanamycin resistance cassette used to delete the *M34* ORF (Figure 5C). These data demonstrate that ΔM34-MCMV is significantly attenuated *in vitro* but clearly replication competent.

### The Analysis of ΔM34HA-MCMV Independently Confirmed the Absence of pM34 and Its Dispensability

**Figure 6:**
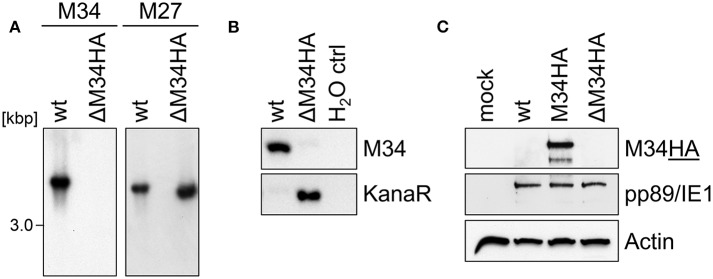
The analysis of ΔM34HA-MCMV independently confirmed the absence of pM34 and its dispensability. **(A)** Generation of a virus mutant harboring a deletion of the *M34HA* ORF (ΔM34HA-MCMV) by replacement with a kanamycin cassette. The specific deletion of the *M34HA* ORF from the BAC was confirmed by Southern blot using probes specific for indicated MCMV genes. **(B)** The absence of *M34HA* from the genome of ΔM34HA-MCMV was confirmed by PCR using DNA extracted from infected cells used for viral stock preparation as template. **(C)** CIM cells were infected with wt-MCMV, M34HA-MCMV, ΔM34HA-MCMV (MOI 0.05), or left uninfected (mock). At 4 d p. i, cells were lysed and immunoblot analysis was performed.

**Figure 7:**
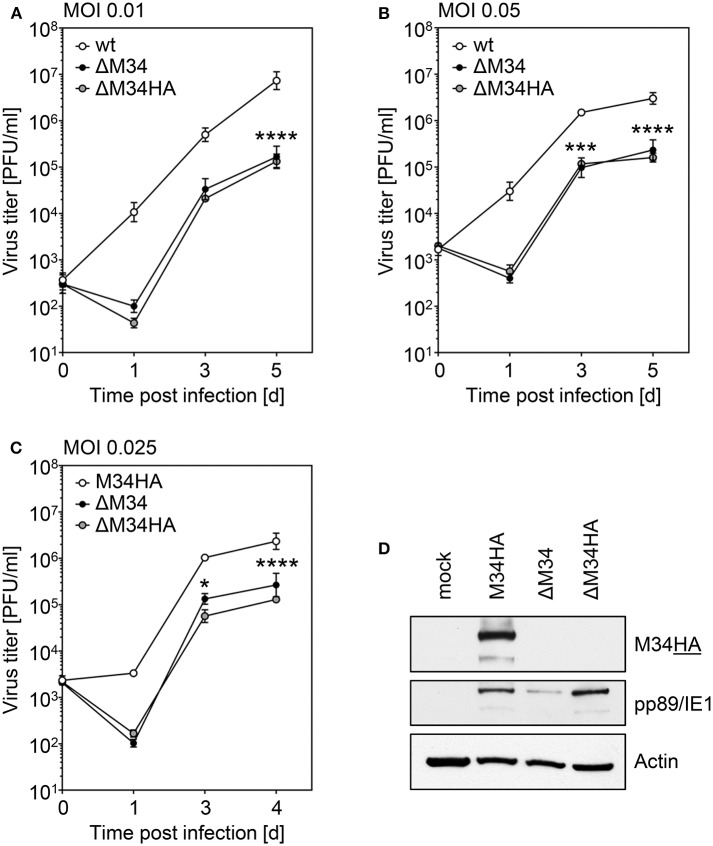
Analysis of ΔM34HA-MCMV verifies that the *M34* gene locus is not essential for MCMV replication. **(A,B)** Primary MNC derived from BALB/c mice were infected with wt-MCMV, ΔM34-MCMV, or ΔM34HA-MCMV with MOI 0.01 **(A)** or MOI 0.05 **(B)**. At indicated time points post infection, virus titers were quantified by standard plaque assay. Each titration was performed in triplicate and depicted as mean values ±SD. ^***^p < 0.001. ^****^p < 0.0001. **(C)** MNC were infected with M34HA-MCMV, ΔM34-MCMV, or ΔM34HA-MCMV with MOI 0.025. Virus titers were quantified as described above. ^*^p < 0.05. ^****^p < 0.0001. Significance was calculated by two-way ANOVA with multiple comparisons. **(D)** Infected cells from D were lysed at 4 d p. i. and analyzed by immunoblotting for indicated proteins.

We are not aware of the availability of pM34-specific antibodies. To formally exclude the presence of pM34 protein, the *M34* deletion was repeated on the background of M34HA-MCMV. The insertion of the kanamycin deletion cassette was directed to replace the entire *M34* coding sequence including the previously inserted HA epitope (Figure 2D). Southern blotting of BAC DNA confirmed the deletion of *M34* (Figure 6A). After virus reconstitution, the absence of *M34* from the genome of replicating ΔM34HA-MCMV was confirmed by PCR (Figure 6B). As shown by immunoblotting, the pM34HA expression was completely abrogated in ΔM34HA-MCMV-infected cells, confirming the successful deletion of *M34* on DNA and protein level as well as in a second and independently generated MCMV mutant (Figure 6C). Upon infection of MNCs, ΔM34HA-MCMV was highly attenuated as compared to wt-MCMV in replication experiments starting with 0.01 (Figure 7A) or 0.05 PFU per cell (Figure 7B). A direct comparison of ΔM34-MCMV and ΔM34HA-MCMV revealed identical replication kinetics of both mutants (Figures 7A,B). The infections were repeated with 0.025 PFU per cell and ΔM34HA-MCMV, ΔM34-MCMV, and M34HA-MCMV as wildtype-like control (Figure 7C). Immunoblotting of lysates prepared from infected cells of this experiment confirmed the absence of pM34HA throughout the attenuated replication (Figure 7D). These data verify that the *M34* gene locus is not essential for MCMV replication.

## Discussion

We provide compelling evidence that *M34*, the MCMV homolog of *UL34*, is not essential for MCMV replication. Replication competence was observed in primary and immortalized MEFs as well as MNCs derived from C57BL/6 and BALB/c mice. This finding was verified by reconstitution of replicating ΔM34-MCMV either harboring or lacking a kanamycin cassette which was used to replace the entire *M34* CDS. Additionally, we generated an M34HA-MCMV expressing an HA-epitope-tagged version of pM34. Using M34HA-MCMV, we showed that pM34HA is expressed with *early*-*late* kinetics and localizes in the nucleus. M34HA-MCMV will enable future studies on protein-protein interactions and protein-DNA interactions in the infection context *in vitro* and *in vivo*. To corroborate the dispensability of pM34 and to formally prove the absence of pM34 protein, the directed deletion was recapitulated using M34HA-MCMV BAC as parental BAC which yielded ΔM34HA-MCMV in which pM34HA protein expression was abrogated.

Successful deletion of *M34* was confirmed by restriction digest, Southern blot, PCR, sequencing, and immunoblot analysis either for the MCMV-BAC or the replicating MCMV mutant. Additionally, we observed a 10- to 100-fold impaired replication of different MCMV mutants lacking *M34*. The independently constructed and reconstituted mutant viruses as well as the respective controls ruled out contaminations with wt-MCMV or the presence of residual parental BAC-derived MCMV. Taken together, we propose to categorize *M34* as highly relevant, protein-coding, non-essential gene.

Previous studies showed that a transposon insertion mutant of *M34* (in which the transposon inserted at position 44,827 of the MCMV genome corresponding to codon 582 of M34) as well as a truncation mutant lacking the coding sequence corresponding to the C-terminal amino acids 548-854 of pM34 were replication competent, while “*following transfection of NIH/3T3 cells with this BAC construct no virus was recovered, despite many repeated attempts the full M34 knock-out BAC could not be reconstituted*” (Baluchova et al., 2008). Intriguingly, the authors stated that “*although characteristic virus CPE was evident in mutant BAC-infected cells BAC-containing virus of sufficient yield to titrate, grow stocks or passage was not achieved*” (Baluchova et al., 2008), suggesting that residual virus replication may have occurred even at that time — a bit like a *viable but nonculturable* (VBNC) state well-known for certain bacteria.

We infer that the choice of the cell line used for reconstitution may have contributed to the different outcome. We successfully reconstituted ΔM34-MCMV in the immortalized MEF cell line CIM which is highly MCMV permissive. We passaged the BAC-transfected cells until replicating virus became evident by CPE and plaque formation. Individual attempts of ours to reconstitute ΔM34-MCMV in NIH/3T3 and primary MEF cells also failed (data not shown). The ΔM34-MCMV replication is reduced 10- to 100-fold as compared to the parental wt-MCMV. This attenuation affects the propagation time necessary to reach sufficient yields for stock preparation following ΔM34-MCMV BAC transfection. Accordingly, we consider our approach to continuously passage the BAC-transfected cells during reconstitution as a main reason for our successful recovery of ΔM34-MCMV. Such a procedure is unfeasible when applying cells which either do not adhere well to surfaces and tend to detach during prolonged culturing especially at higher confluency (such as NIH/3T3) or become senescent (such as non-immortalized primary cells). Although we have not carefully compared the reconstitution efficacy of our CIM cell line with NIH/3T3 cells and primary MEFs in side-by-side experiments, the herein described method and cell line may be worthwhile to try in the future, especially in case MCMV mutants are expected to exhibit severe replication impairments.

As reported, Baluchova et al. tried to reconstitute the ΔM34-MCMV mutant in NIH/3T3 (ATCC^®^ CRL-1658™) cells which is a commonly used stable mouse fibroblast cell line derived from NIH/Swiss mice (Jainchill et al., 1969). Although we do not consider this very likely, we cannot formally rule out that genetic differences between mouse strains (e.g., BALB/c and C57BL/6 vs. the outbred strain NIH/Swiss) may render *M34* indispensable for reconstitution from a transfected BAC.

According to Brocchieri et al. and Tang et al., the coding sequence of *M34* overlaps with the genes *m33.1, m34.1*, and *m34.2* (Brocchieri et al., 2005; Tang et al., 2006). Ectopic expression of a Flag-epitope-tagged pm34.2 localizes to mitochondria, while “*a deletion in m34.2 resulted in small plaques and a decrease in virus production of nearly 2 orders of magnitude*” (Tang et al., 2006). The full deletion of *M34* as done by Baluchova et al. and by us necessarily also eliminates *m33.1* and *m34.1*. Thus, our data also indicate that neither *m33.1* nor *m34.1* are essential for MCMV replication. As result of the *M34* deletion, only the first 66 codons of pm34.2 are retained followed by an artificial C-terminus comprising 14 amino acids before a Stop codon is reached. Thus, the impaired replication of ΔM34-MCMV may in part result from the C-terminal truncation and alteration of pm34.2 and/or the deletion of *m33.1* and *m34.1*.

The gene *UL34* is essential for HCMV replication, while its homolog *M34* is non-essential for MCMV replication. During HCMV infection, pUL34 acts as transcriptional repressor for viral genes such as *US3* and *US9*. Additionally, pUL34 binds the *oriLyt* region (LaPierre and Biegalke, 2001; Liu and Biegalke, 2013) and enhances the efficiency of *oriLyt*-dependent DNA replication of plasmids (Slayton et al., 2018). Site-directed mutagenesis of the pUL34 binding sites in the *oriLyt* of HCMV reduced HCMV replication (Slayton et al., 2018). Since the minimal functional domain for transcriptional repression maps to amino acids 22 to 243, which constitute the most conserved region of pUL34-like proteins, it is tempting to speculate that the impaired replication of ΔM34-MCMV mutants may also result from the lack of pM34 DNA-binding and gene repression and/or its effect on the *oriLyt*. The genome of MCMV Smith (GenBank: GU305914) harbors 4 fully conserved pUL34 binding sites (consensus sequence AAACACCGTK). Taking the previously described binding of pUL34 to related DNA motifs (Slayton et al., 2018) into account, we identified 10 potential pUL34 binding sites (consensus sequence AAACRCCGTB) in the MCMV genome. However, none of those resides within or is localized very close (<1 kb) to the MCMV major IE promoter (MIEP) [identified in Dorsch-Häsler et al. (1985) or the MCMV *oriLyt;* identified in Masse et al. (1997)], suggesting that either the DNA motif recognized by pM34 differs from the pUL34 DNA binding motif, or that other genes are affected.

Taken together, our findings indicate that *M34* (and the overlapping ORFs *m33.1*, and *m34.1*) are not essential for MCMV replication, enabling future studies on the function of the pUL34 homolog pM34 in the context of infection *in vitro* and *in vivo*.

## Data Availability Statement

All datasets generated for this study are included in the article/supplementary files.

## Ethics Statement

The generation of primary MEF and MNC is described in the cited publication Le-Trilling and Trilling, 2017.

## Author Contributions

ME and VL-T did research. ME, VL-T, and MT analyzed data. VL-T and MT supervised the project. All authors wrote and revised the manuscript.

## Conflict of Interest

The authors declare that the research was conducted in the absence of any commercial or financial relationships that could be construed as a potential conflict of interest.
